# The Tumor Microenvironment in Tumorigenesis and Therapy Resistance Revisited

**DOI:** 10.3390/cancers15020376

**Published:** 2023-01-06

**Authors:** Kevin Dzobo, Dimakatso A. Senthebane, Collet Dandara

**Affiliations:** 1 Wound and Keloid Scarring Research Unit, Hair and Skin Research Laboratory, Division of Dermatology, Department of Medicine, The South African Medical Research Council, Groote Schuur Hospital, Faculty of Health Sciences, University of Cape Town, Anzio Road, Observatory, Cape Town 7925, South Africa; 2 Division of Medical Biochemistry and Institute of Infectious Disease and Molecular Medicine, Department of Integrative Biomedical Sciences, Faculty of Health Sciences, University of Cape Town, Anzio Road, Observatory, Cape Town 7925, South Africa; 3 Division of Human Genetics, Department of Pathology, Faculty of Health Sciences, Institute of Infectious Disease and Molecular Medicine, University of Cape Town, Anzio Road, Observatory, Cape Town 7925, South Africa; 4 The South African Medical Research Council-UCT Platform for Pharmacogenomics Research and Translation, Department of Pathology, Faculty of Health Sciences, University of Cape Town, Anzio Road, Observatory, Cape Town 7925, South Africa

**Keywords:** tumor microenvironment, stromal cells, immune cells, ECM, cancer hallmarks, hypoxia, exosomes, drug resistance, targeted therapy

## Abstract

**Simple Summary:**

Tumors are not masses of cancer cells alone but made up of cancer cells, other cells including fibroblasts, macrophages, endothelial cells, as well as secreted factors, blood vessels and the extracellular matrix (ECM). This comprehensive review presents new findings on the role of each component of the tumor cell surroundings and the effect on the success of cancer drugs. We show in this paper that the tumor cell’s surroundings are not simply ‘bystanders’ but are actively involved in tumor growth and can cause resistance to treatment. Initially, cells and ECM around tumor cells do not promote their growth but over time, tumor cells ‘convert’ their surroundings to promote their growth. An increase in tumor size means tumor cells must overcome a lack of oxygen and nutrients, be able to remove waste and form secondary tumors. A better knowledge of tumor cells and their surrounding means better drugs for tumor cells and their surroundings.

**Abstract:**

Tumorigenesis is a complex and dynamic process involving cell-cell and cell-extracellular matrix (ECM) interactions that allow tumor cell growth, drug resistance and metastasis. This review provides an updated summary of the role played by the tumor microenvironment (TME) components and hypoxia in tumorigenesis, and highlight various ways through which tumor cells reprogram normal cells into phenotypes that are pro-tumorigenic, including cancer associated- fibroblasts, -macrophages and -endothelial cells. Tumor cells secrete numerous factors leading to the transformation of a previously anti-tumorigenic environment into a pro-tumorigenic environment. Once formed, solid tumors continue to interact with various stromal cells, including local and infiltrating fibroblasts, macrophages, mesenchymal stem cells, endothelial cells, pericytes, and secreted factors and the ECM within the tumor microenvironment (TME). The TME is key to tumorigenesis, drug response and treatment outcome. Importantly, stromal cells and secreted factors can initially be anti-tumorigenic, but over time promote tumorigenesis and induce therapy resistance. To counter hypoxia, increased angiogenesis leads to the formation of new vascular networks in order to actively promote and sustain tumor growth via the supply of oxygen and nutrients, whilst removing metabolic waste. Angiogenic vascular network formation aid in tumor cell metastatic dissemination. Successful tumor treatment and novel drug development require the identification and therapeutic targeting of pro-tumorigenic components of the TME including cancer-associated- fibroblasts (CAFs) and -macrophages (CAMs), hypoxia, blocking ECM-receptor interactions, in addition to the targeting of tumor cells. The reprogramming of stromal cells and the immune response to be anti-tumorigenic is key to therapeutic success. Lastly, this review highlights potential TME- and hypoxia-centered therapies under investigation.

## 1. Methodology

**Figure 1:**
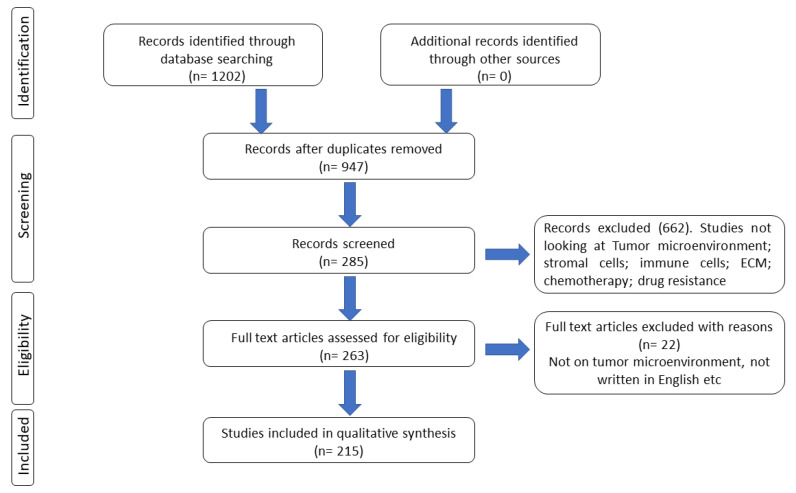
Selection of manuscripts used in the production of this review manuscript.

We retrieved relevant published manuscripts via an electronic search on Embase, Scopus, PubMed and Web of Science using keywords including tumor microenvironment; stromal cells; immune cells; extracellular matrix (ECM); cancer hallmarks; hypoxia; chemotherapy; multi-drug resistance and targeted therapy. This search yielded a rich source of data on the role of tumor microenvironment in tumorigenesis and therapy resistance (Figure 1). We removed duplicate articles and only full articles were included in compiling this review.

## 2. The Tumor Microenvironment in Brief

**Figure 2:**
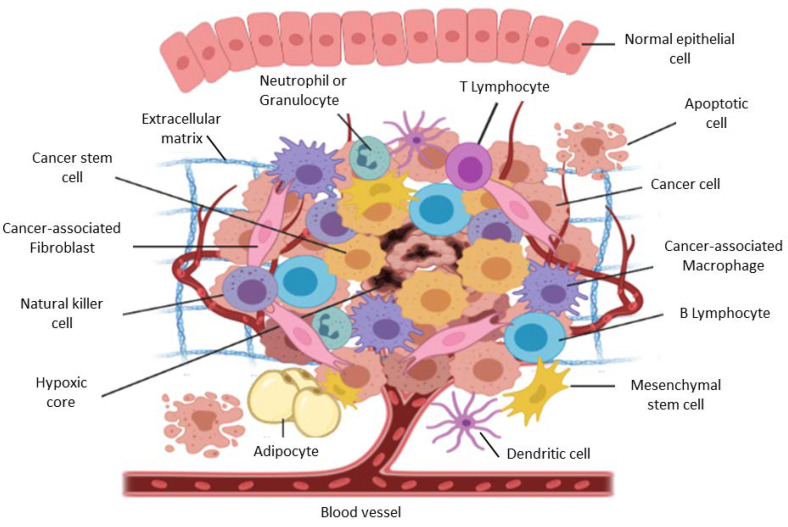
The tumor microenvironment components and their contribution during tumorigenesis.

It is universally accepted that cancer has major hallmarks including the presence of genomic instability and mutations, unrestricted growth, the evasion of growth suppressors, resisting cell death, enhanced inflammation, enhanced metabolism, and the ability to promote angiogenesis, invasion, and metastasis [1,2]. It is also scientifically accepted that tumors are more than just tumor cells and include recruited stromal cells and the non-cellular component, the ECM (Figure 2) [3,4,5,6]. Stromal cells and the ECM are active participants during tumorigenesis, starting as anti-tumorigenic during the initial stages to being pro-tumorigenic over time and contributing to the attainment of specific cancer hallmarks [3,4,5,6]. Thus, the study and understanding of cancer and tumorigenesis now extends beyond tumor cells to include the stromal cells and the ECM, which make up the tumor microenvironment (TME) [3,5,6,7,8,9,10,11,12,13,14,15,16,17,18]. Stromal cells include normal fibroblasts, cancer associated fibroblasts (CAFs), cancer associated macrophages (CAMs), mesenchymal stem cells (MSCs), inflammatory cells and endothelial cells [3,7,11,13,17,19,20]. Beside the contribution of the TME during tumorigenesis and metastasis, the TME and common features including hypoxia also play a critical role in therapy resistance [4,6,8,9,14,16,18]. Cell-cell and cell-ECM interactions involve a myriad of biomolecular factors, such as growth factors, cytokines, enzymes, and chemokines. In addition, exosomes and apoptotic bodies are shown to play roles in promoting tumorigenesis and drug resistance [21]. This review provides a comprehensive description of how the TME, characterized by hypoxia, contribute to tumorigenesis and therapy resistance, and presents ways to reprogram cells and factors to increase therapy efficacy.

## 3. Biological Functions of Stromal and Immune Cells within the Tumor Microenvironment

### 3.1. Cancer Associated Fibroblasts

**Figure 3:**
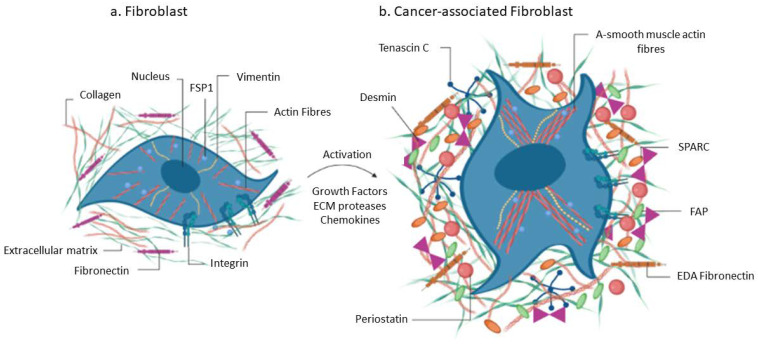
Normal fibroblasts are initially anti-tumorigenic within the TME but over time become activated into CAFs and contribute to tumorigenesis through increased synthesis of factors and the ECM.

Reports have shown that resident and recruited fibroblasts are part of the TME, where they contribute during tumorigenesis and in drug resistance [3,4,5,7,12,17,19,20]. Initially, fibroblasts are anti-tumorigenic as they are involved in the synthesis of the ECM, which surrounds and isolates tumor cells from normal tissue during the early stages of tumorigenesis [5,6,22]. Over time, a subpopulation of activated fibroblasts, referred to as cancer associated fibroblasts, obtain a myofibroblastic phenotype characterized by the increased synthesis of ECM and the release of pro-tumorigenic factors (Figure 3) [6,23]. Similar to myofibroblasts linked to fibrosis, the CAFs are perpetually activated and promote tumorigenesis via the release of factors, the activation of pro-tumorigenic signaling, angiogenesis, microRNA, and cytokines [24,25,26,27,28]. At each stage of tumorigenesis, CAFs continue to produce and interact with various TME components including the ECM, cytokines, and growth factors.

Together with several other stromal cells including CAMs and MSCs, CAFs release factors such as TGF-β and cytokines involved in ECM remodeling, the promotion of tumor cell proliferation, the suppression of immune response, the recruitment of MSCs as well as the induction of angiogenesis [3,5,6,16,17,29,30]. For example, TGF-β, from both tumor cells and CAFs, has been shown to promote tumor cell proliferation and to induce EMT transition [31,32,33,34,35,36]. TGF-β overexpression is correlated with poor prognosis in prostate cancer, colorectal cancer, and hepatocellular carcinoma [37,38,39]. In addition to the expression of TGF-β, CAFs also express vascular endothelial growth factor (VEGF) and platelet derived growth factor (PDGF), and this allow their involvement in tumor metastasis [40,41]. CAF-derived interleukin-6 (IL-6) activation of the Janus kinase (JAK)-signal transducer and activator of transcription (STAT) (JAK-STAT) signaling pathway leads to increased TGF-β signaling, promoting tumor growth and metastasis [42,43,44]. In addition, increased CAFs within the TME and synthesized CXC chemokines correlated with low patient survival in various cancers including colorectal cancer and esophageal cancer [45,46,47,48]. Several investigations also show that CAF-derived matrix metalloproteases (MMPs) participate in tumor cell migration and invasion through the creation of ‘matrix highways’ after ECM molecules degradation [49,50,51,52,53]. Using a cell-derived ECM, Senthebane and colleagues demonstrated that fibroblast-derived MMPs contribute towards cancer cell migratory and invasive behavior [5].

**Figure 4:**
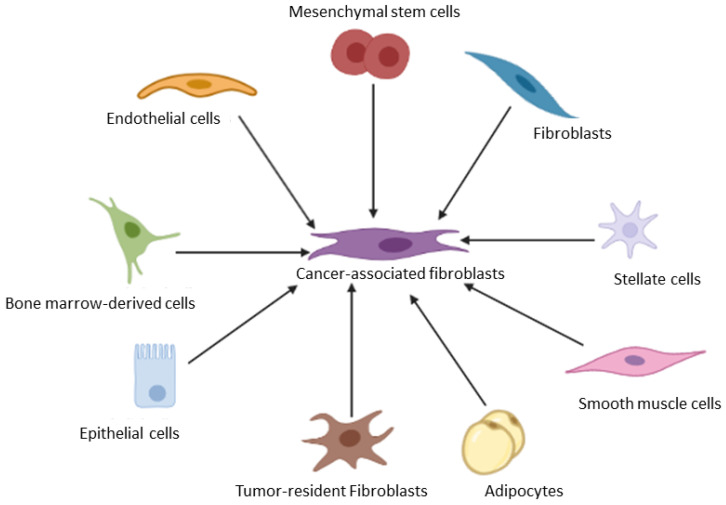
Cancer associated fibroblasts are diverse in origin. The origin of CAFs range from tissue resident fibroblasts, pericytes, endothelial cells to mesenchymal stem cells.

Mounting reports indicate that CAFs originate from different cells and therefore display complex heterogeneity (Figure 4) [16]. Tissue-resident fibroblasts contribute to most CAFs within the TME in addition to other stromal cells such as stellate cells, bone marrow derived- and tissue adult derived-MSCs, pericytes and endothelial cells (Figure 3) [54]. In the case of an injury, the activation of tissue-resident fibroblasts and stellate cells in the liver, for example, reversibly transform these cells into a myofibroblast phenotype characterized by the elevated expression of α-SMA [54]. Many studies have shown the involvement of growth factors including fibroblast growth factor 2 and TGF-β signaling in the transformation of stromal cells into myofibroblastic cells or CAFs [5,24,31,42]. These myofibroblastic cells are the activated fibroblasts responsible for enhanced ECM synthesis in liver cancers. Several studies have also shown that fibrocytes are present in blood [55]. Barth and colleagues demonstrated the presence and the role of CD34+ fibrocytes in invasive ductal carcinoma [56]. Besides breast cancer, the same authors also demonstrated a role for fibrocytes in pancreatic and cervical cancer [57]. Overall, the increased levels of CAFs within the TME is associated with tumor relapse and poor prognosis in various cancers.

Another potential origin of CAFs is epithelial cells. Epithelial cells near cancer cells can undergo epithelial-to-mesenchymal transition (EMT) and end up as CAFs [58]. Epithelial cancers may display elevated levels of CAFs that drive tumorigenesis [17,59]. Epithelial cells lose the normal cell-cell adhesive abilities and gain migratory abilities. Endothelial cells can undergo endothelial-to-mesenchymal transition (EMT), transforming these cells into CAFs [60]. CAFs originating from epithelial and endothelial cells produce CAF markers such as S100A4 [58,60]. Both adipocytes and pericytes can undergo trans-differentiation into CAFs [61]. It is important to note that while CAFs are pro-tumorigenic, studies also indicate that CAFs can act in an anti-tumorigenic manner [16,62]. Only recently, is a clear and well-defined picture of CAFs and their role in tumorigenesis emerging.

CAFs heterogeneity means numerous subgroups exist with contrasting phenotypes and functions within the TME [63,64]. Reports also show that CAFs heterogeneity is linked to stage of tumor development [65]. ECM remodeling and stromal cell transformations during different stages of tumorigenesis can lead to CAFs being genetically unstable [66,67]. Thus, CAFs co-evolve with tumor cells during tumorigenesis. The initial anti-tumor activity of stromal cells becomes ‘tumor-promoting’ activity over time [5,6]. Various signaling cascades modulate CAFs activation and activity and these include the lysophosphatidic acid and TGF-β family ligands which influence serum response factor (SRF) and SMAD transcription factors activities, respectively, to promote the expression of the activated fibroblast marker α-SMA [68]. A co-culture of cancer cells and fibroblasts demonstrate the promotion of CAF activation in breast cancer via the Notch signaling [69]. Furthermore, inflammatory modulators including interleukin-1β (IL-1β) can induce NF-_K_B activation in CAFs [70]. CAF markers include FAP, PDGFRα/β, tenascin C, vimentin, desmin, CD90 and podoplanin (PDPN) (Table 1) [71]. CAFs heterogeneity means that there is no universal marker and early studies utilized α-SMA and FAP-alpha [71]. A combination of these markers is the ideal means to identify CAFs. Other markers include α-SMA, vimentin and CD10. The expression of α-SMA is not exclusive to CAFs as other cells such as smooth muscle cells and pericytes express the same marker [63]. CAFs found in several cancers, such as breast and pancreatic cancers, express high levels of α-SMA and vimentin [54,72].

Targeting CAFs, with their significant heterogeneity, involves reversal of the transformation from normal fibroblasts into CAFs. Reports indicate that the use of microRNA can achieve such de-activation or reprogramming of CAFs into normal fibroblasts [83,84,85]. De-differentiation of CAFs into quiescent cells is another strategy under consideration [86].

### 3.2. Cancer Associated Endothelial Cells

New blood vessel formation during tumorigenesis is initiated by endothelial cells and these cells constitute the innermost layer of blood vessels [87]. The usually thin vascular endothelium separates blood from tissues in addition to delivering important nutrients, ions, and water [88]. The vascular endothelium is also important in carrying away all toxic metabolic waste products. Immune cells are also carried to tumors via the blood stream. Whilst diffusion is responsible for oxygen supply and carbon dioxide removal during the initial stages of tumorigenesis, increase in the size of tumor will require increased supply of oxygen as well as removal of metabolic waste [89]. As the tumor increase in size, a hypoxic core is formed, activating the tumor to form new blood vessels to supply much-needed nutrients and oxygen [90,91]. Vascular networks are formed as a result of the action of various transcription factors induced by hypoxia. The transcription factors induced by hypoxia act on endothelial cells which release growth factors, such as epidermal growth factor (EGF), and PDGF to form new blood vessels [92,93]. Old blood vessels can also sprout and form new branching vessels. Beside growth factors, endothelial cells also release proteins required for the formation of basement membranes. Due to the unregulated release of cytokines and growth factors, blood vessel formation is not proper within a tumor. This results in ‘makeshift’ blood vessels that are leaky [94]. Being responsible for new blood vessel formation makes endothelial cells important for cancer cell migration and metastasis. As the blood vessels within tumors are leaky, cancer cells can easily invade new tissues and intravasate into blood vessels to be transported to new sites [95]. Endothelial cells can also undergo ‘endothelial to mesenchymal transition’ to become cancer associated fibroblasts as they are very plastic [96,97]. Various growth factors, including TGF-β, are known to be involved in this transition [98]. Cancer associated endothelial cells promotes tumorigenesis by being immunosuppressive, growth factor synthesis and the enhanced migratory behavior of tumor cells [99,100]. Cancer associated endothelial cells also aid immunosuppressing myeloid cells’ infiltration into tumors. Reports show that cancer associated endothelial cells can modulate anti-tumor immunity via the disruption of cytotoxic T cell infiltration, whilst at the same time allowing immunosuppressive cells to move into the tumor [14,101]. Cancer associated-endothelial cells also demonstrate enhanced angiogenic ability leading to increased drug resistance versus normal endothelial cells [102,103].

### 3.3. Cancer-Associated Macrophages

In the human body, macrophages, mostly originating from circulating monocytes, participate in various processes from clearing infections and wound healing, as well as the repair of tissues [104]. As part of the innate immune system macrophages respond to the presence of pathogens by presenting antigens and carrying out phagocytosis [105]. M1 macrophages are the predominant type of macrophages during the initial stages of tumorigenesis, as they participate in phagocytosis of pathogens and antigen presentation [106]. A tumor is sometimes referred to as a ‘wound’ that does not heal. Thus, within the tumor microenvironment, the M2 macrophages are present and actively participate in suppressing the immune system and wound healing [107]. Deep inside the tumor, la ack of oxygen and various cytokines are known to promote the M2 type of macrophages [107,108]. The infiltration of tumors with macrophages occur throughout the process of tumorigenesis and macrophages can account up to a third of the mass of the tumor at some stages. Reports indicate that an elevated levels of macrophages within tumors are associated with low survival rates in various cancers [109,110]. This is attributed to macrophages’ promotion of angiogenesis via release of various cytokines and thus enhance formation of new blood vessels. Recent data also show that CAMs play key roles in chemoresistance to drugs such as paclitaxel and 5-fluorouracil [111,112,113,114,115,116]. Furthermore, CAMs have been shown to promote CSCs tumorigenic capacity as well as their therapeutic resistance via increased enzyme synthesis (cytidine deaminase) involved in drug metabolism [112,113,114].

### 3.4. Cancer-Associated Neutrophils

When an infection occurs, circulating leukocytes, and specifically neutrophils, provide the first line of defense against pathogens [117]. Within the tumor microenvironment, neutrophils can have both pro- and anti-tumorigenic properties [118]. During the initial stages of tumorigenesis, recruited neutrophils release various cytokines including IL6 thereby inducing inflammation [119,120]. This causes tumor cells to undergo apoptosis. Neutrophils also release reactive oxygen species that induce apoptosis in tumor cells [104]. In later stages of tumorigenesis, neutrophils release various growth factors such as VEGF involved in angiogenesis, and therefore promotes tumorigenesis through new blood vessel formation [121,122]. Neutrophils are also involved in ECM remodeling via the production of matrix metalloproteases (MMPs) [123]. MMPs are also actively involved in promoting tumor cell invasion and eventual metastasis via the degradation of ECM molecules [124]. Cancer-associated neutrophils have been shown to contribute towards the attainment of acquired cancer drug resistance via their ability to suppress the immune system, enhancement of angiogenesis, as well as enhancing tumor cell proliferation [117,125]. Cancer associated neutrophils also activate various signaling cascades that prevent the proper functioning of many cancer drugs such as immune checkpoint blockers and common cytotoxic drugs. In combination with standard therapies, drugs targeting cancer associated neutrophils can sensitizes tumor cells to drugs and prevent drug resistance and relapse [121,125].

### 3.5. T Cells

Various populations of T cells have been identified within the tumor microenvironment at various stages of tumor development [126]. Specific T cell populations have specific receptors used in antigen identification. For example, cytotoxic T cells with specific receptors identify abnormal antigens expressed on tumor cells and their attachment to tumor cells leads to the destruction of the cells [126,127]. Cytotoxic T cells also play a key role in preventing formation of new blood vessels via the release of the pleiotropic cytokine interferon-gamma [128]. Thus, cytotoxic T cells demonstrate anti-tumorigenic behavior within the tumor microenvironment [129]. Another population of T cells found within the tumor microenvironment are the CD4+ T cells. CD4+ T cells are mainly involved in immune responses within the tumor microenvironment and over time differentiate into several cells [130]. For example, CD4+ T cells can become T-helper 1 cells, which participate in inflammation induction and their presence within various tumors is linked to increased patient survival [131,132,133]. Another T cell type found within tumors is the regulatory T cells. Regulatory T cells participate in suppressing inflammation and anti-tumor immune responses [134,135,136,137]. Regulatory T cells releases interleukin-2, which controls the function of natural killer cells [138,139,140]. Furthermore, regulatory T cells secrete various growth factors and cytokines and advertently supports tumorigenesis [139,140].

### 3.6. B Cells

B cells are responsible for antibody production in the body as well as secretion of various cytokines [141,142,143,144]. B cells are mostly localized at the periphery of tumors and within lymph nodes near the tumor site [142,144]. Thus, few B cells are found within tumors [142,144]. The main function of B cells during tumorigenesis is their close relationship with T cells, allowing T cells to act against tumor cells. B cells act as antigen presenting cells to T cells [145,146,147,148]. B cells are also involved in secretion of anti-tumorigenic cytokines such as IFN-γ [145,146,147,148]. However, several studies also show that B cells are pro-tumorigenic in some tumors [149,150,151]. It has been shown that regulatory B cells produce various cytokines including IL-10 and TGF-β that promote immune suppression via their effects on macrophages and T cells [152,153,154].

### 3.7. Natural Killer Cells

Natural killer cells are able to destroy cells infected with viruses in blood [8,155,156]. Two functional sub-categories of natural killer cells have been identified: those that directly kill tumor cells; whilst another sub-category produces inflammatory cytokines [8,133,155]. Inflammation will lead to the accumulation of various immune cells involved in tumor cell killing. By seeking and destroying tumor cells within the bloodstream, natural killer cells are important in preventing metastasis and formation of secondary tumors [157,158,159]. Both natural killer cells and innate natural killer cells use both adhesion and cytokine receptors to identify their cellular targets and in so doing can spare normal healthy cells [160,161]. Within tumors, natural killer cells are less efficient at killing tumor cells. Reports indicate that both natural killer cells and innate natural killer cells are able to detect ‘stress’ or biological changes in host tissues and the cells can activate innate and adaptive immune cells within the TME [161,162,163,164]. Reports indicate that natural killer cells may express multi-drug resistance-like activity, and this can be inhibited through the use of drugs such as verapamil or solutol HS-15 [165,166,167].

### 3.8. Dendritic Cells

The function of dendritic cells is mostly to recognize and capture antigens as well as present them to T cells [168,169,170]. Dendritic cells are mostly found within lymph nodes where they participate in T cell response to specific pathogen infection [171,172]. Depending on the prevailing environment within tumors, dendritic cells can be both anti- and pro-tumorigenic [170]. The over-production of pro-tumorigenic growth factors and cytokines can lead to dendritic cells tolerating the presence of tumor cells and act to prevent an immune reaction [170]. Tumors have been shown to exploit dendritic cells. For example, reports show that local dendritic cells may be conditioned by tumor cells to form suppressive T cells, leading drug resistance [173,174].

### 3.9. Stellate Cells

Found in the liver and the pancreas, stellate cells originated from mesenchymal tissue and are mostly involved in promoting tumorigenesis via differentiation into myofibroblasts [175,176,177]. Injury to the liver and pancreas induce stellate cell differentiation into myofibroblasts, after which they synthesize enormous quantities of ECM molecules and growth factors including VEGF [178,179,180]. The development of a tumor, akin to ‘wound healing’ induce stellate cells differentiation into myofibroblasts. One major function of stellate cells is the accumulation of vitamin A in lipids droplets [181,182,183]. The lipid droplets are then utilized during ECM synthesis and production of MMPs. Tumor cell-derived TGF-β is known to be involved in the activation of hepatic stellate cells into myofibroblast in liver cancer. Both liver cancer and pancreatic cancer tend to be associated with fibrosis. Quiescent pancreatic stellate cells are involved in remodeling of the ECM via MMPs synthesis and ECM protein synthesis [184]. Once activated, pancreatic stellate cells secrete various biomolecules leading to their increased migratory behavior and proliferation. Various reports demonstrated the involvement of stellate cells in tumorigenesis [185,186,187]. For example, a classic study by Hessmann and colleagues showed that pancreatic stellate cells traps drugs such as gemcitabine and this reduces the efficacy of the drug during treatment [188].

### 3.10. Adipocytes

Two cell types, adipocytes, and white adipose tissue, constitute the adipose tissue [189]. Energy storage as well as maintenance of energy balance in the body is the function of adipocytes or fat cells. Given the high energy required by tumor cells during tumor initiation and progression, it is not surprising therefore that adipocytes play a key role in this process [189]. Adipocytes have been shown to secrete various biomolecules from growth factors, enzymes to cytokines [190,191]. The secretion of enzymes including MMPs leads to ECM remodeling, allowing tumor cells to migrate and metastasize. Obesity is considered a high-risk factor in many cancers with close to half cancer patients being obese for example in breast and ovarian cancers [192]. Reports show that white adipose tissue is linked to an increased risk of cancers and the formation of secondary tumors in lungs, for example [193]. Organs with a high number of adipocytes include the breast and these cells have been shown to be pro-tumorigenic [194]. As tumor cells require a lot of energy, adipocytes can be induced to undergo lipolysis, which converts lipids into fatty acids that can be used by tumor cells during tumorigenesis [189,195]. Furthermore, adipocytes secrete various hormones including leptin that promotes tumor cell proliferation and migration as well as the recruitment of immune cells to the TME [196]. Adipose-derived adult stem cells, which can differentiate into different cell lineages, also come from adipose tissue. These stem cells have the ability to enhance inflammation within the TME and thus are pro-tumorigenic [197,198]. It is possible that adipose-derived stem cells can differentiate into cancer-associated stromal cells such as CAFs.

### 3.11. Mesenchymal Stem Cells

Important for the maintenance of healthy tissue and the repair of tissue in the case of injury, mesenchymal stem cells, or mesenchymal stromal cells, are able to differentiate into cell types such as osteoblasts, and chondrocytes [199,200]. This differentiation ability is the reason why MSCs recruited to tumors can transform into various tumor associated cells. Reports indicate that beside resident fibroblasts differentiation into CAFs, recruited MSCs can also be transformed into CAFs [4,16,17,20,24]. Whilst resident fibroblasts may initially have an anti-tumorigenic phenotype, it is reported that over time all fibroblasts are pro-tumorigenic [5]. During the initial stages of tumorigenesis, fibroblasts synthesize large quantities of ECM proteins, in what appear to be an attempt at isolating the tumor from the rest of the tissue [5]. An increase in ECM synthesis also causes the stiffening of tissue. An increase in tissue stiffness has been associated with tumorigenesis [201]. In later stages of tumorigenesis, MSCs demonstrate immunoregulatory effects by contributing to the dampening of the anti-tumor immunity [202,203]. Importantly, the differentiation of MSCs into CAFs has long-lasting effect with regard to the promotion of tumorigenesis as CAFs will continue to synthesize and release various factors needed by tumor cells. Our earlier study clearly demonstrated the involvement of MSCs in CAFs differentiation and the release of TGF-β, for example [6].

### 3.12. Pericytes

Pericytes have multiple roles within the tumor microenvironment including covering endothelial cells along the surface of the endothelium, in the remodeling of the basement membrane during tumorigenesis and the formation of new blood vessels [94,204]. Pericytes have also been involved in immunoregulatory process through the activation of immune cells such as lymphocytes, and in phagocytosis [205,206]. Although clinical trials targeting pericytes involvement in angiogenesis has been carried out, results so far are not promising. Some reports even show that targeting pericytes leads to more tumor cells metastasizing [207,208]. For example, targeting pericytes in animal models of breast cancer resulted in aggressive pulmonary tumor process [209]. It has been postulated that pericytes may display heterogeneity and there is need to target the correct pericyte subpopulation with a specific phenotype to stop tumorigenesis [210,211,212]. Reports indicate that pericytes can cause resistance to vemurafenib and sorafenib in thyroid cancer and the mechanism involved occur via the TGF-β signaling [213]. Other reports show that pericytes participate in tumorigenesis via the promotion of angiogenesis [214,215].

## 4. The Extracellular Matrix

**Figure 5:**
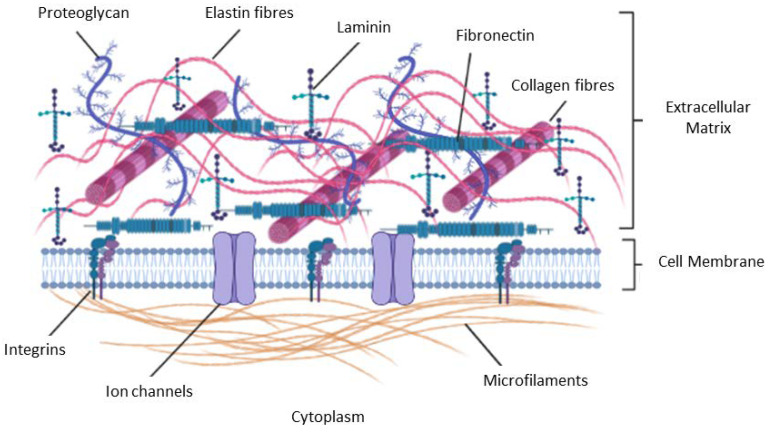
Components of the ECM include glycoproteins, collagens, proteoglycans, and polysaccharides. Collagens and glycoproteins are ligand for integrins and play key roles in tumor cell signaling necessary for survival.

One key component of the TME is the ECM. Forming the structural part of the TME, the ECM is located under the epithelial layer surrounding the connective tissue cells [216,217]. CAFs are the main source of ECM components. It is made up of many macromolecules including vitronectin, collagens, proteoglycans, and glycoproteins (e.g., fibronectin, laminin) (Figure 5) [216]. Its composition is always changing depending on the stage of tumorigenesis [218], and this is facilitated by enzymes such as cathepsins, lysyl oxidase (LOX), MMPs, and their inhibitors [219]. In solid tumors, the ECM can constitute about half of the tumor mass (desmoplastic tumors) and has been linked to poor patient survival [220].

The elasticity and rigidity of the ECM promote tumorigenesis via integrin signaling [221]. Changes in ECM composition and elasticity influence many aspects of tumorigenesis varying from cancer cell growth, survival and therapy resistance [221]. Collagen, the most abundant ECM molecule in tumors [221], provides structural support to tumor cells and regulating other processes such as tumor cell adhesion, supporting chemotaxis and migration. Enhanced levels of type I collagen also increase ECM stiffness and promote tumorigenesis in the process [221]. Enzyme-linked changes in ECM composition and levels facilitate tumor cell migration via the creation of ‘pores’ allowing tumor cells to invade surrounding tissues and travel to distant tissues and organs [222]. Increased collagen production and the resulting stiffness influence integrin signaling and tumor cell survival [222].

Importantly, the ECM presents a physical hindrance to drug distribution within tumors [223]. In most cases, this physical hindrance as well as the sequestration of drugs through direct binding to ECM molecules contributes to the development of drug resistance in many solid tumors [224]. Furthermore, various reports show that the ECM is key to tumor vascularization [225]. New blood vessel formation is important to tumorigenesis. As production of ECM molecules such as collagen increases, the resulting increased ECM density causes a decrease in vascularization. A stiff ECM compresses blood vessels, limiting the flow of drugs and oxygen within the TME [225,226]. The lack of enough oxygen within tumors influence vascularization via the activation of HIF-1α. HIF-1α promotes chemoresistance via activation of MDR1 expression in hypoxic colon cancer, for example [227,228]. Lastly, the ECM can sequester various growth factors and cytokines that can promote tumorigenesis such as TGF-β, VEGF and PDGF.

## 5. Vascular Networks

**Figure 6:**
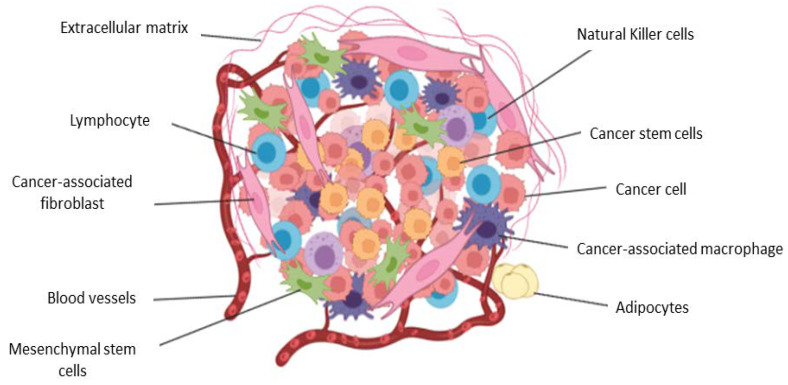
The vascular networks are important during tumorigenesis. Supply of oxygen and removal of carbon dioxide and other metabolic waste products is achieved by the blood vessels. Leaky blood vessels also allow tumor cells to migrate to other tissues and organs.

Tumor cells require supplies of oxygen and nutrients to maintain their uncontrolled growth [229]. This is achieved through the vascular networks that allows gaseous exchange and the removal of toxic waste from the tumor (Figure 6) [8,230]. A major hallmark of cancer is the process of angiogenesis. The tumor microenvironment becomes hypoxic as the tumor continue to grow as the vasculature cannot supply oxygen to all cells within the TME [8]. New blood vessels formed from pre-existing ones are ‘leaky’ and convoluted [7,231]. Similar to the growth of tumor cells, which is uncontrolled, blood vessel formation continues unabated with no proper control, resulting in a complex structure. Leaky vessels also help tumor cells to migrate to other tissues and organs to form secondary tumors, as well as contribute to the ineffective distribution of drugs within the TME. Lymphatic vessels also provide a ‘throughfare’ through which tumor cells can migrate to other sites [232]. Indeed, lymph nodes have been shown to be the common sites through which tumor cells migrate to other tissue and organs [233]. In many cancers, lymph node metastases are linked to poor prognosis [234,235,236]. Once in the lymph nodes, cancer cells can easily migrate to other organs and tissues and in many cases lymph nodes metastases must be treated together with solid tumors for successful therapy.

## 6. Hypoxia within the TME

A hallmark of the unregulated proliferation of tumor cells is the unavailability of oxygen or hypoxia and nutrients in some parts of a growing solid tumor [237,238]. Synthesis of new blood vessels via angiogenesis does not occur fast enough to provide oxygen to rapidly growing tumor cells. The result is tumors with some regions having less than 2% oxygen levels, thus are hypoxic [238,239]. Importantly, angiogenesis within a growing tumor leads to dysregulated vasculature and oxygenated blood is not supplied to all regions. Tumor cells within hypoxic regions obtain a different phenotype to those in regions properly supplied with oxygenated blood, are more aggressive and become resistant to commonly used drugs [240]. Indeed, oxygen gradients within solid tumors is a common feature. Tumor cells within hypoxic regions also express elevated levels of hypoxia-inducible factor alpha (HIF-1α), with three isoforms having been found in mammals [241]. HIFs play central roles in tumorigenesis in which they influence hypoxia-induced gene expression and metabolism [242]. For example, HIF-1 is especially important in tumor cell response to therapy [243]. HIF-1α also enhances the activities of transcriptional factors including Twist and Snail, leading to increased endothelial-to-mesenchymal transition (EMT) [244,245]. By modulating collagen synthesis and collagen fiber alignment as well as integrin-ECM interactions within the TME, HIF-1α also aid tumor cell migration and metastasis [246,247]. In addition, due to a lack of oxygen, tumor cells within hypoxic regions of TME divide slowly, thus can circumvent common drugs targeting rapidly dividing tumor cells.

As a tumor grows, de novo angiogenesis leads to the formation of leaky blood vessels leading to an increase in interstitial fluid pressure [95,248]. Furthermore, leaky blood vessels aid tumor cell metastasis as tumor cells can easily escape the blood vessels with discontinuous endothelium. Various reports documented that cells within hypoxic TME region also promote immunosuppression. For example, cancer-associated macrophages of the M2 type have been found in hypoxic regions [249,250]. The immunosuppressive properties of CAMs are well documented. HIF-1α can modulate the behavior of myeloid-derived suppressor cells within the hypoxic regions of TME [251]. Hypoxia also cause the TME to be acidic and under these conditions T cells are not able to perform their cytotoxic functions [252]. Further data show that hypoxia can induce the over-expression of various proteins involved in drug efflux [239]. Reports show that the blocking of HIF-1α expression can reverse drug resistance in cancers [253,254]. Drug resistance can also emanate from tumor cells altering their metabolism and avoiding apoptosis. Hypoxia can also induce autophagy, which can lead to multi-drug resistance [255]. Overall, hypoxia within the TME can be used as an independent prognostic factor in cancers and predicts poor outcomes [256,257]. Thus, novel strategies must target tumor hypoxia together with various components of the TME.

## 7. Exosomes and Exosomal miRNAs in Tumor Microenvironment

Ranging in size from 30 to 200 nm, exosomes play key roles in cellular communication between tumor cells and stromal cells and are secreted into the extracellular space by cells regularly [258]. The contents of exosomes depend on their origin, with stromal cell-derived exosomes containing various growth factors, cytokines and other signaling molecules that can impact tumor cell behavior as well as cell-cell interactions [259]. In most cases, the contents of exosomes promote tumorigenesis via impacting processes such as angiogenesis, migration, and metastasis [260]. Reports indicate that tumor cells under conditions of low oxygen and nutrients produce increased levels of exosomes and leads to alterations of stromal cells into pro-tumorigenic cells including CAFs and CAMs [21,261]. Tumor cell-derived exosomes also have the ability to prepare some tissue-specific cells for colonization by tumor cells [262,263].

Importantly, exosomes are key to transporting microRNAs [21]. Stromal cells can alter microRNAs (miRNAs) expression in both tumor cells and stromal cells [261]. The alteration of miRNA expression can be induced by tumor and stromal cell interactions through the release of auto- and paracrine factors [86,260]. For example, microRNA-122 from breast cancer cells has been shown to reprogram normal cell metabolism by reducing the uptake of glucose by lung cells, in preparation for lung colonization [264,265,266]. This will make sure there are enough nutrients for metastatic breast tumor cells upon lung colonization. Delineating miRNAs functions within the TME can lead to new therapeutic targets identification. Exosomes are useful as diagnostic biomarkers as well as therapeutic targets [267]. Exosomes are stable within the circulatory system and their contents can be used for diagnosis purposes and can predict tumor metastasis accurately [268,269]. Given their many functions during tumorigenesis, reports indicate that the abrogation of exosome production can inhibit tumorigenesis [21,270]. The suppression of tumor-derived exosomes uptake through the use of heparin resulted in decreased metastatic ability of oral squamous cell carcinoma [271].

In terms of cancer treatment, exosomes can be used to deliver drugs as they are non-toxic and biodegradable [272]. Ligands specific for certain tumors can be expressed on the surface of the exosomes so as to direct them to specific tumor cells [273,274]. Such tumor cell-specific exosomes can then deliver therapeutic siRNA or drugs, for example, to kill cancer cells [275].

## 8. Advances in Therapeutic Targeting of TME

Great improvements have been brought to cancer treatment through combinations of various drugs and immunotherapy in the past few years. Chemotherapy, used mostly as the first line of cancer treatment, target rapidly growing cancer cells, and tends to be broad in its focus [276,277]. Whilst cancer is initially caused by changes in genes, its progression is associated with major biological and metabolic changes that over time negatively affect bodily functions [278,279]. By specifically targeting sub-populations of cancer cells within the TME including CSCs, improvements have been made in cancer treatment [3,18]. In addition, the introduction of immunotherapy and specifically immune checkpoint blockade such as PD1 that targets several immune cells within the TME brought remarkable success in cancer treatment [280]. Immune checkpoint inhibitors are antibodies or drugs that block proteins called checkpoints from immune system cells including T cells as well as some cancer cells [281,282]. Programmed death ligand 1(PDL-1) on cancer cells and the programmed death 1 (PD-1) on normal healthy cells are important in the maintenance of immune responses [7]. When cancer cell PDL-1 interacts with PD-1 on normal cells, this prevents the immune reaction of the normal cells to the presence of tumor cell. Checkpoint inhibitors prevent the interaction between PDL-1 and PD-1 and thus allow normal cells to activate the immune reaction to the presence of cancer cells. Currently, checkpoint inhibitors have been clinically proven for various cancers including renal cell carcinoma, colon cancer and lung cancer, among others [7,283,284]. The major advantages of checkpoint inhibitors include low toxicity and being able to reduce tumor mass efficiently [285,286]. Normally, these checkpoints prevent the immune responses from being too strong and this impact T cells’ ability to kill cancer cells [287,288]. Importantly, the identification of biomarkers can lead to the grouping of patients that can benefit from specific drugs and therapies.

New therapies also include the prevention of new blood vessel formation. Tumorigenesis is a process that depends on the constant supply of oxygen and nutrients to growing tumor cells [94]. Furthermore, metabolic waste must be removed, without which the microenvironment becomes toxic even for tumor cells. Thus, the prevention of angiogenesis through the use of anti-angiogenic drugs including those neutralizing growth factors such as VEGF, decoy receptors for growth factors is an appealing strategy under intense investigation. Small molecule inhibitors of several factors released within the TME including AMD3465 can prevent stromal cell-derived factors from being pro-tumorigenic [289,290]. Antagonists of integrins can prevent cell-cell and cell-ECM interactions within the TME, increasing cancer cell response to drugs in the process [291]. ECM proteins play key roles in cancer cell migration, invasion and survival and thus blocking ECM protein interactions with their major surface receptor, integrins, can influence drug efficacy and tumor progression [226,292,293]. For example, the combination of celengitide, an integrin antagonist, and temozolomide, resulted in improved antitumor activity against malignant melanoma [294]. The inactivation of HIF-1α has been shown to enhance the effect of carboplatin on tumor cell proliferation and thus can be used as a hypoxia-centered therapy [295,296]. Tumor acidification has been identified to be a major characteristic of tumor progression as well as a regulator of tumor response to drugs [7]. The acidification of the TME by hypoxia also reduces some drug effectiveness as this depends on the surrounding pH. The pH of the TME also regulates cellular metabolic rates and can influence tumor cell metastatic abilities [297,298]. An adjustment of TME pH can therefore be used to enhance or decrease the efficacy of drugs [299]. Drugs that can be activated in the hypoxic regions of tumors have been suggested. These hypoxic pro-drugs can be activated into cytotoxic drugs by enzymes found within the hypoxic regions of tumors. For example, TH-302 is a hypoxic pro-drug utilized together with gemcitabine in the treatment of pancreatic cancer, which is highly hypoxic with oxygen levels averaging around 0.7% [300,301]. Various signaling cascades important in hypoxia including the unfolded protein response are appealing targets to treat solid tumors characterized by hypoxia [302,303]. Other strategies to avoid hypoxia-induced changes to drug effectiveness make use of nanoparticles to deliver drugs directly to tumor cells. Other strategies to inhibit hypoxia-mediated HIF response if to use small-interfering RNA. Detailed reviews on TME-centered therapies has already been published elsewhere [7,8,304,305,306].

As expected, therapy resistance is a major problem when these strategies are used. Combination therapy involving the use of two or more anti-tumor strategies results in better responses. More research is needed, including evaluating the efficiency of these strategies before these strategies are commonplace in clinics.

## 9. Conclusions

The treatment of cancer, ranging from the use of surgery, chemotherapy, radiotherapy, and recently introduced immunotherapy, have all had limited success when used alone. In most cases, combination therapy is the best strategy to use for successful treatment. However, therapy resistance develops as tumor cells are heterogenous and plastic in nature, and tumor cells can convert a non-supporting ‘anti-tumorigenic’ environment into a ‘pro-tumorigenic’ environment. The contribution of the tumor microenvironment to tumorigenesis, metastasis, and the development of therapy resistance, is of note. Thus, it is important to delineate the role played by various TME components in tumorigenesis, metastasis, and therapy development. This review discusses the identification of predictive, prognostic biomarkers via the analysis of TME components and how this reveals the complexity of tumor biology, as well as lead to the development of targeted therapies for specific cancers and patients. Importantly, the recruitment of non-tumorigenic cells and non-cellular components by tumor cells for their benefit, allows tumorigenesis to proceed without hindrances. Stromal cells and immune cells are reprogrammed by tumor cells to release various factors that favor tumor cell growth and survival. The hypoxic microenvironment has been noted to play key roles in tumorigenesis and drug resistance. Understanding the processes involved in regulating hypoxia can lead to new therapeutic targets. In this regard, exosomes have been identified as useful as diagnostic and therapeutic tools by revealing tumor-derived secretome and can deliver drugs to tumor cells, respectively. Currently, combination therapy targeting various components of the TME can lead to the best results during treatment.

## Figures and Tables

**Table 1:** Markers for cancer-associated fibroblasts.

CAFs Marker	Description and Function of Protein	Effect within TME
α-SMA	Actin isoform: cellular contraction and maintenance of structure	Promote tumor cell proliferation; involved in immunosuppression [73,74,75]
Tenascin-C	Extracellular matrix glycoprotein: cell migration; wound healing	Impeding drug delivery; protect tumor cells [76,77]
Vimentin	Type III intermediate filament protein: cell migration; cell structure maintenance	Tumor cell migration and invasion [25,34,74,75]
PDGFRα/β	Protein tyrosine kinase receptor: cellular signaling	Macrophage polarization; angiogenesis [16,78]
FAP	Membrane-bound gelatinase: protease activity; ECM remodeling	Angiogenesis, macrophage polarization; immunosuppression; metastasis [16,17,79]
GPR77	Complement component 5a receptor 2: Activation of complement; promote inflammation	Maintains tumor cell stemness; Drug resistance [80]
Caveolin-1	Scaffolding protein within caveolar membranes: maintains cellular structure and signaling	Low caveolin-1 linked to poor prognosis [81,82]
